# Associations of TG/HDL Ratio with the Risk of Prediabetes and Diabetes in Chinese Adults: A Chinese Population Cohort Study Based on Open Data

**DOI:** 10.1155/2021/9949579

**Published:** 2021-07-08

**Authors:** Rongpeng Gong, Yuanyuan Liu, Gang Luo, Wenjing Liu, Ziqi Jin, Zixin Xu, Zheng Li, Lixin Yang, Xiaoxing Wei

**Affiliations:** ^1^Medical College of Qinghai University, Xining, Qinghai 810016, China; ^2^Endocrinology Department, Qinghai Provincial People's Hospital, Xining, Qinghai 810007, China; ^3^College of Eco-environmental Engineering, Qinghai University, Xining, Qinghai 810016, China; ^4^Center for Reproductive Medicine of the First Affiliated Hospital of Zhengzhou University, Zhengzhou, Henan 450000, China; ^5^Shaanxi University of Chinese Medicine, Xi'an, Shaanxi 712046, China

## Abstract

**Background:**

In the global chronic diseases, type 2 diabetes shows a significant upward trend, and there are more people before prediabetes (impaired glucose tolerance). Many patients with impaired glucose tolerance and undiagnosed diabetes do not know that their glucose metabolism system has been in a state of disorder. Every year, about 5% to 10% of prediabetics develop diabetes. One of the important achieving factors may be the increase in blood lipids. However, it is not clear whether the triglyceride/high-density lipoprotein ratio is associated with impaired glucose tolerance and diabetes in the Chinese population. Therefore, we investigated the relationship between triglyceride/high-density lipoprotein and impaired glucose tolerance and diabetes in the Chinese population.

**Methods:**

We conducted a retrospective cohort study using data from the health screening program. The study included 116,855 participants from 32 locations in China, all of whom were adults over 20. Participants measured fasting blood glucose levels at each visit and collected information about their diabetes history. Impaired glucose tolerance was diagnosed as fasting blood glucose ≥6.00 mmol and self-reported diabetes mellitus. The patient was measured on the date of diagnosis or on his last visit (whichever comes first).

**Results:**

The results showed that, after adjusting the potential confounding factors, the ratio of TG/HDL was positively correlated with the occurrence of prediabetes and diabetes, and there was a saturation effect. The inflection points were 1.04 and 1.33, respectively. The effect value and 95% confidence interval before and after the inflection point of impaired glucose tolerance patients were 1.57 and (1.42, 1.73) and 1.03 and (1.01, 1.05), respectively. The effect value and 95% confidence interval before and after the inflection point in patients with diabetes were 2.07 and (1.80, 2.39) and 1.08 and (1.04, 1.12).

## 1. Background

Type 2 diabetes is a global chronic disease. In recent years, type 2 diabetes has been on the rise among chronic diseases worldwide [1]. As the most common chronic disease, diabetes puts a tremendous financial strain on both patients and healthcare systems. Simultaneously, some related studies have found that patients with diabetes have a worse prognosis and a higher mortality rate after being infected with COVID-19 [2–5]. According to the International Diabetes Federation (IDF), there are about 425 million people (20–79 years old) with type 2 diabetes (T2DM) worldwide in 2019. By 2045, this number is expected to rise to 642 million, of which 140.2 million live in Asia [6–8].

Prediabetes is the development of impaired glucose tolerance that does not meet the diagnostic criteria for type 2 diabetes [9]. In addition to type 2 diabetes, more people are currently prediabetic (impaired glucose tolerance), and these people are potentially at risk for type 2 diabetes [9, 10]. Many patients with prediabetes and untested and diagnosed diabetes are unaware of the disorder in their glucose metabolism and do not take preventive measures. As a result, approximately 5% to 10% of patients with prediabetes progress to diabetes each year [10].

In recent years, patients with impaired glucose tolerance and type 2 diabetes have tended to be younger [1, 11]. Younger patients with impaired glucose tolerance and type 2 diabetes tend to have a poor prognosis and are associated with an increased risk of cardiovascular disease and microvascular complications [12]. The decline in the age of the initial onset of prediabetes and type 2 diabetes is unclear and complex. Some people speculate that this is related to the modern lifestyle. The increased probability of high-fat diet intake may be one reason for prediabetes, younger diabetic, and the number of people with diabetes [11, 13–19]. Therefore, it is of great significance to predict or screen out prediabetic patients (especially in the young population) to control the total number of diabetic population.

Related research shows that the onset of diabetes and age seem to have a critical connection [20]. Aging is itself a risk factor for diabetes: for every additional ten years a person spends, the risk of developing diabetes goes up by 50–70% [20–22]. However, the increase in the population's high-fat diets weakens the protective effect of age on diabetes incidence [23]. A high-fat diet can increase the amount of TG synthesis in the liver, lower the HDL relatively, and then increase the ratio of TG/HDL [24–27].

The population of diabetes in China ranks first globally, and there is an increasing trend year by year [8, 28–30]. This is closely related to China's rapid economic development and the change of the dietary structure. Among them, the high-fat and high-protein diet significantly increased during the same period. At present, some studies have reported the relationship between the TG/HDL ratio and the incidence of diabetes. For example, studies in Japan, South Korea, and the United States have shown that TG/HDL is an independent risk factor for the incidence of diabetes. However, the effect sizes of these results are inconsistent [31–33]. This may be due to the significant differences between ethnic groups. At the same time, these studies only analyzed the relationship between TG/HDL and diabetes and did not include patients with the prediabetic stage. Therefore, it is of great significance to analyze whether the ratio of TG/HDL is correlated with the incidence of prediabetes and diabetes. In this study, based on the published data, we analyzed whether TG/HDL is a risk factor for prediabetes and diabetes in the Chinese population.

## 2. Methods

### 2.1. Study Design and Participants

**Figure 1:**
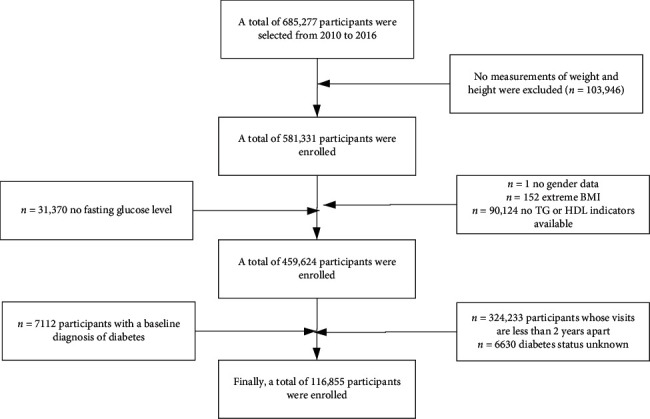
Flowchart of patient selection.

Our data come from Dryad public data (https://datadryad.org/stash), extracted by Chinese researchers (Chen et al.) from a computerized database set up by Ruiz Medical Group, covering 32 regions in China and 11 cities (Shanghai, Beijing, Shenzhen, Nanjing, Suzhou, Changzhou, Hefei, Wuhan, Chengdu, Guangzhou, and Nantong). The data include the medical records of all participants who received health checkups from 2010 to 2016. Participants initially included were at least 20 years of age and had undergone at least two health checkups between 2010 and 2016 (*n* = 685,277). At the time of inclusion, participants were excluded if they had no available weight and height measurements (*n* = 103946), no available information on gender (*n* = 1), an extreme BMI value (<15 kg/m^2^ or >55 kg/m^2^) (*n* = 152), no available TG or HDL indicators (*n* = 90124), or no available fasting glucose value (*n* = 31370). Participants with less than two years between visits (*n* = 324,233), participants diagnosed with diabetes at enrolment (2997 confirmed by self-report and 4115 diagnosed with fasting glucose ≥6.0 mmol), and participants with unknown diabetes status at follow-up (*n* = 6630) were further excluded. In the end, a total of 116,855 participants (62,868 men and 53,987 women) were included in the analysis (Figure 1). The queue entry is defined as the date of the first access. Compared to the individuals excluded from this analysis, the individuals included in the analysis were similar in age (42.1 vs. 41.9 years), had a similar body mass index (23.2 vs. 23.3 kg/m^2^), and had a relatively higher proportion of males (54.8% vs. 52.1%). Participants were asked to fill out a detailed questionnaire to assess demographics, lifestyle, medical history, and family history of chronic diseases at each visit to the health screening center. Height, weight, and blood pressure are measured by trained staff. Weight is measured without shoes and wearing light clothing, with weight measured to one decimal point and height measured to one decimal point. BMI is calculated by dividing weight in kilograms by height in meters squared. Blood pressure is measured with a standard mercury sphygmomanometer. Fasting venous blood samples were collected after a minimum of 10 hours of fasting for each examination. Serum triglyceride (TG), total cholesterol (TC), low-density lipoprotein cholesterol (LDL-C), and high-density lipoprotein cholesterol (HDL-C) were measured by the Beckman 5800 automatic analyzer. Glucose oxidase assay was used to measure blood glucose levels on a Beckman 5800 automatic analyzer.

The study was a retrospective cohort study. Participants were divided into two ratio groups based on saturation effects, with impaired glucose tolerance being divided into *N*1 (TG/HDL < 1.04) and *N*2 (TG/HDL ≥ 1.04), and diabetes is divided into *N*1 (TG/HDL < 1.33) and *N*2 (TG/HDL ≥ 1.33).

### 2.2. Ascertainment of Incident Prediabetes and Diabetes

The diagnostic criteria of impaired diabetes were fasting blood glucose ≥6.00 mmoL/L and self-reported diabetes at follow-up. The diagnostic criteria of diabetes were fasting blood glucose ≥7.00 and self-exposed diabetes. Patients with prediabetes were reexamined on the date of diagnosis or on their last visit (whichever appears first).

### 2.3. Statistical Analysis

Statistical analysis was performed using R statistical software (R 4.0.3). Bilateral *P*, values less than 0.05 were considered statistically significant. Data from the descriptive analysis were reported as mean (SD) or median (IQR) or scale. Cox proportional regression model was used to analyze the interaction between TG/HDL, impaired glucose tolerance, and underage diabetes, sex, smoking, alcohol consumption, and other groups. Cox proportional hazard regression model was used to estimate the relationship between TG/HDL ratio and impaired glucose tolerance and diabetes incidence (HR) (95% CI). The *N*1 group was selected as the control group, and the effect of the *N*2 group on the risk of prediabetes and diabetes was analyzed (HR, 95% CI). Covariates in the multivariate model included age, sex, smoking status, alcohol consumption status, BMI, biochemical markers, and a family history of diabetes. The effects of age on TG/HDL, impaired glucose tolerance, and diabetes incidence were further investigated. Because of the saturation effect between TG/HDL and impaired glucose tolerance and the risk of diabetes, we describe the risk scores of different groups in two segments, representing the increase of each unit of TG/HDL, the increased risk of impaired glucose tolerance, and diabetes. Curative effects of age, BMI, gender, and TG/HDL interactions were assessed in a Cox model.

## 3. Result

### 3.1. Baseline Characterization

In this study, the median follow-up time of 3.1 years, a total of 116,855 participants were included in the final participants of impaired glucose tolerance and diabetes is respectively 9773 and 2689, including impaired glucose tolerance (6536 cases, 66.9%), diabetes (1579 cases, 72.7%) of male participants which was higher than female impaired glucose tolerance (3237 cases, 33.1%), and diabetes (594 cases, 27.3%) of participants. The mean age and standard deviation of participants with diabetes (55.7 ± 12.6 years) were higher than those of participants with impaired glucose tolerance (54.2 ± 13.0 years) and nondiabetic participants (43.8 ± 12.8 years). The proportion of smoking in the total population and the proportion of people with impaired glucose tolerance and diabetes were 20.4%, 30.6%, and 34.7%, respectively, and these proportions gradually increased. The proportion of participants with alcohol consumption, impaired glucose tolerance, and diabetes (2.7% to 4.5%) increased. Those with diabetes had a BMI of 26.3 ± 3.4, higher than those with prediabetes (25.4 ± 3.3) and those without diabetes (23.3 ± 3.3) (Table 1).

### 3.2. Univariate Analysis

This study analyzed the effects of age, gender, BMI, alcohol consumption, smoking, and some biochemical indicators on prediabetes and diabetes in the Chinese population. We found that women have much lower rates of prediabetes and diabetes than men. Compared to men, women had a 44 percent lower risk of prediabetes, with an effect value (HR) of 0.58 percent and a 95 percent confidence interval (95% CI) for (0.56, 0.61). The effect HR and 95% confidence interval for diabetes in women were 0.44 and (0.4, 0.49), respectively. Compared with participants without a family history of diabetes, participants with a family history of diabetes had a higher incidence, with effect size and 95% confidence interval of 1.06 and (0.94, 1.2) for preonset diabetes. The effect size HR and 95% confidence interval for diabetes were 1.24 and (0.98, 1.57), respectively. The effect values of HR and 95% confidence intervals for age were 1.05 and (1.05, 1.05) and 1.06 and (1.06, 1.06) for prediabetes and diabetes, 1.18 and (1.17, 1.18) and 1.24 and (1.23, 1.25) for BMI, and 1.36 and (1.33, 1.4) and 1.33 and (1.25, 1.40) for LDL, respectively. HDL may be a protective factor for the onset of prediabetes and diabetes, and the practical value of HR and 95% confidence interval for participants with higher HDL were 0.6 and (0.53, 0.68) and 0.58 and (0.50, 0.66), respectively, for prediabetes and diabetes (Table 2).

### 3.3. Curve Fitting and Saturation Effect Analysis

**Figure 2:**
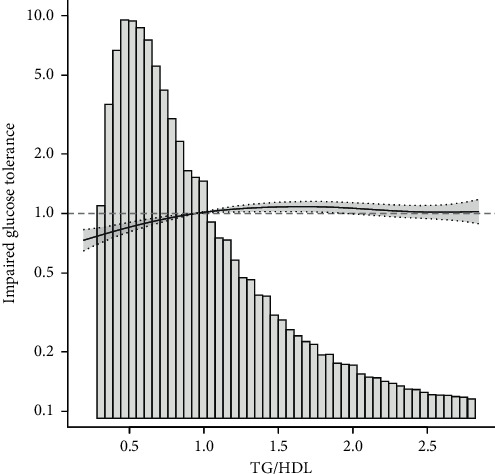
Association between TG/HDL and impaired glucose tolerance.

**Figure 3:**
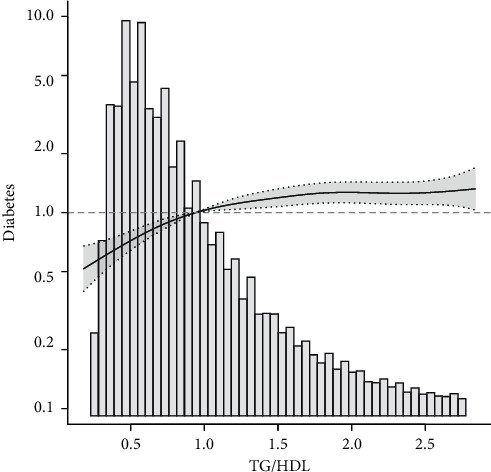
Association between TG/HDL and diabetes mellitus.

**Figure 4:**
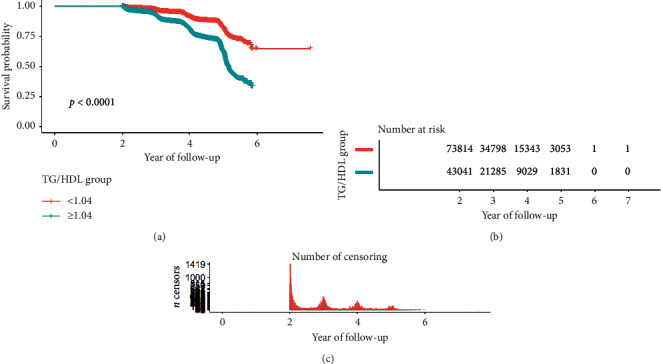
Survival analysis of impaired glucose tolerance with TG/HDL ratio.

**Figure 5:**
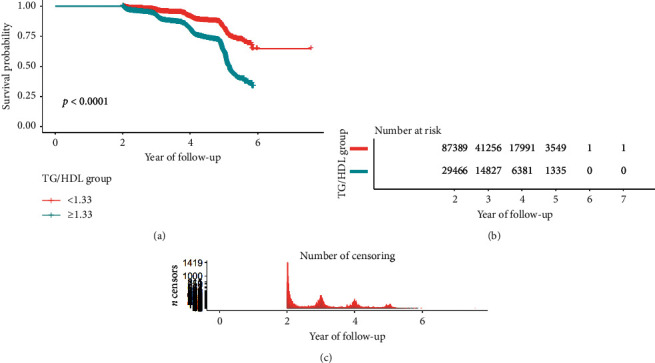
Survival analysis of diabetes mellitus with TG/HDL ratio.

This study drew a smoothing fitting curve to analyze the relationship between TG/HDL ratio and prediabetes and diabetes mellitus in the Chinese population (Figures 2 and 3). The results showed that TG/HDL had a saturation effect on both prediabetes and diabetes mellitus (Tables 3 and 4), and the inflection points were 1.04 and 1.33, respectively. Prediabetes: when TG/HDL <  1.04, there was a linear relationship between TG/HDL and prediabetes, the effect value and 95% confidence interval were 1.57 and (1.42, 1.73), and *P* < 0.001, effect value was very significant; when TG/HDL ≥ 1.04, the risk of prediabetes did not increase with the increase of TG/HDL (Table 3). When TG/HDL <  1.04 and TG/HDL ≥ 1.04, the effect difference between the two stages was 0.64 (0.57, 0.71) (Table 3). Diabetes mellitus: when TG/HDL <  1.33, TG/HDL had a linear relationship with diabetes mellitus, the effect value HR and 95% confidence interval were 2.07 and (1.80, 2.39), and *P* < 0.001 effect value was very significant. When TG/HDL ≥ 1.33, TG/HDL also had a linear relationship with diabetes, but it was weaker than <1.33. The effect value HR and 95% confidence interval were 1.08 and (1.04, 1.12), respectively (*P* < 0.001). The effect difference between TG/HDL <  1.33 and TG/HDL ≥ 1.33 was 0.52 (0.45, 0.61) (*P* < 0.0001) (Table 3). In this study, it was found that when TG/HDL ≥ 1.04, the risk of prediabetes was much higher than that when TG/HDL <  1.04 (Figure 4), and when TG/HDL ≥ 1.33, the risk of diabetes was much higher than that when TG/HDL < 1.33 (Figure 5).

### 3.4. Multivariate Regression Analysis

Table 5 shows the association between the TG/HDL ratio and the probability of prediabetes after adjusting for potential confounders.

We have built four regression models to analyze the results, while model 1 was adjusted according to age, gender, and BMI. In model 2, family history of diabetes, smoking, drinking, and hypertension were added on the basis of model 1, and in model 3, ALT, AST, BUN, LDL, FPG, and TC were added on the basis of model 2. The results showed that the ratio of TG/HDL was related to the occurrence of prediabetes in all models. In the population with TG/HDL <  1.04, the effect value HR and 95% confidence interval of the unadjusted model were 7.71 and (7.05, 8.43). The effect value HR and 95% confidence interval adjusted for model 1 were 2.37 and (2.15, 2.62), *P* < 0.001, and the difference was statistically significant. The effect value HR and 95% confidence interval adjusted for model 2 were 2.29 and (2.07, 2.53), *P* < 0.001, and the difference was statistically significant. The effect value HR and 95% confidence interval adjusted according to model 3 were 1.57 and (1.42, 1.73), *P* < 0.001, and the difference was statistically significant. In the population with TG/HDL ≥ 1.04, the effect HR and 95% confidence interval adjusted according to the complete model were 1.00 and (0.98, 1.02), *P*=0.9558.

The effect value had no clinical value, and the difference was not statistically significant.  Table 6 shows the relationship between the TG/HDL ratio and the probability of developing diabetes after adjusting for potential confounders. The results showed that adjusted model 3, model 2, and model 1 suggested that the ratio of TG/HDL was related to the occurrence of diabetes. In the population with TG/HDL < 1.33, the effect value HR and 95% confidence interval of the unadjusted model were 6.60 and (5.86, 7.43), respectively. The effect value HR and 95% confidence interval adjusted for model 1 were 2.46 and (2.16, 2.81), respectively, *P* < 0.001, and the difference was statistically significant. The effect value HR and 95% confidence interval adjusted for model 2 were 1.14 and (1.11, 1.16), respectively, *P* < 0.001, and the difference was statistically significant. The effect value HR and 95% confidence interval adjusted according to model 3 were 2.28 and (1.99, 2.61), respectively, *P* < 0.001, and the difference was statistically significant. In the population with TG/HDL ≥ 1.33, the unadjusted model effect value (HR) and 95% confidence interval (95% CI) were 1.23 and (1.21, 1.25), respectively. The effect value HR and 95% confidence interval adjusted for model 1 were 1.13 and (1.10, 1.16), respectively, *P* < 0.001, and the difference was statistically significant. The effect value HR and 95% confidence interval adjusted for model 2 were 1.14 and (1.11, 1.16), respectively, *P* < 0.001, and the difference was statistically significant. The effect value HR and 95% confidence interval adjusted according to model 3 were 1.12 and (1.09, 1.15), respectively, *P* < 0.001, and the difference was statistically significant.

### 3.5. Subgroup Analysis

**Figure 6:**
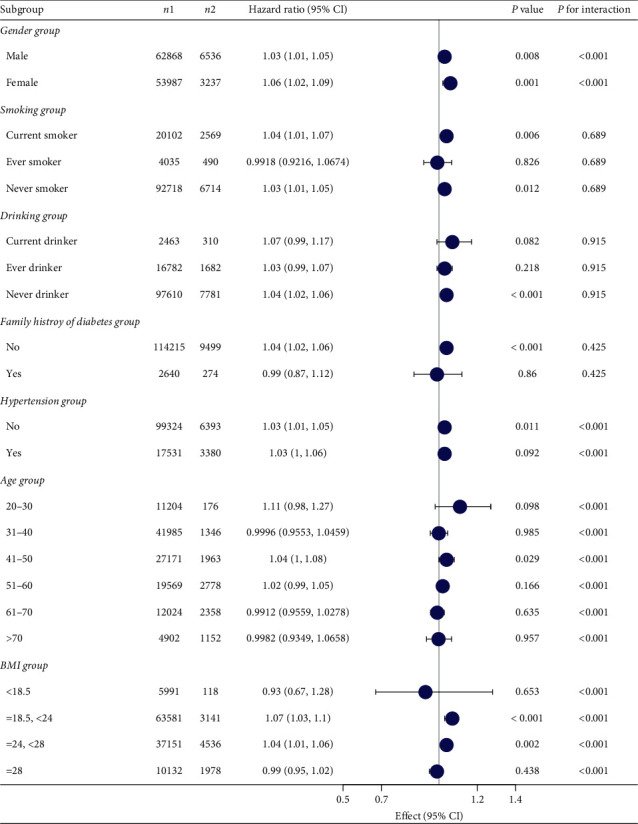
Subgroup analysis of the association between TG/HDL and impaired glucose tolerance.

**Figure 7:**
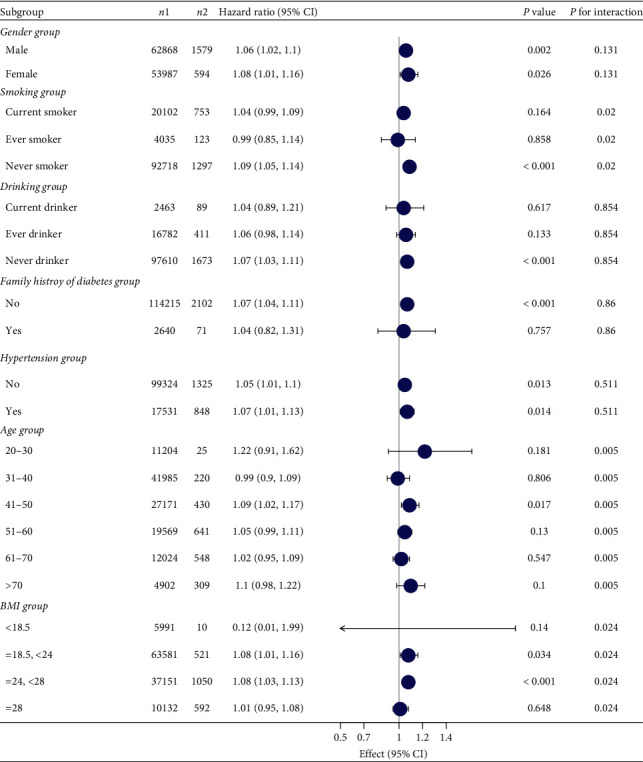
Subgroup analysis of the association between TG/HDL and diabetes mellitus.

Figure 6 shows that the relationship between the ratio of TG/HDL and the incidence of prediabetes remained stable in all subgroups.  Figure 7 shows that the relationship between the ratio of TG/HDL and the incidence of diabetes remained stable in all subgroups. Among age, sex, hypertension, and BMI, the relationship between TG/HDL ratio and prediabetes and diabetes gradually weakened with age. The interaction between TG/HDL and prediabetes and diabetes was most excellent at a BMI of 18.5–24.

## 4. Discussion

Our results showed that, after adjusting for potential confounding factors, the ratio of TG/HDL was positively correlated with the occurrence of prediabetes and diabetes mellitus, with saturation effect, and the inflection points were 1.04 and 1.33, respectively. Prediabetes: the effect value and 95% confidence interval before and after the inflection point were 1.57 and (1.42, 1.73) and 1.03 and (1.01, 1.05), respectively. Diabetes mellitus: effect sizes and 95% confidence intervals before and after the inflection point were 2.28 and (1.99, 2.61) and 1.15 and (1.11, 1.18), respectively. At the same time, subgroup analysis was conducted according to the inflection point, which could better help us find the relationship between TG/HDL and impaired glucose tolerance. TG/HDL interacted with impaired glucose tolerance, diabetes, age, gender, and BMI. With the increase of age, the association of TG/HDL gradually weakened. The association between TG/HDL and prediabetes reached the maximum when BMI was 18.5–24; that is, the effect value of TG/HDL was more significant in the younger population and was more significant in the population with a BMI of 18.5–24, which accounted for the majority of the study, but had a low proportion of diabetes. Therefore, it was more meaningful to pay attention to this group of people.

In previous studies, TG/HDL was often used to evaluate insulin resistance [34–38]. Recent studies have found a correlation between TG/HDL and the incidence of diabetes [39–42]. However, these studies did not include patients with prediabetes. When participants develop insulin resistance, it does not lead directly to the onset of diabetes in the patient. However, it gradually leads to impaired glucose tolerance, and in most cases, these individuals will develop diabetes [9]. In contrast, on the basis of diabetes, our study further included participants with abnormal glucose metabolism and clinically elevated fasting blood glucose into the analysis and found that TG/HDL is not only associated with diabetes but also related to impaired glucose tolerance. Our findings are helpful for the early detection of diabetes and timely measures.

Compared with another study based on the same database, TG/HDL and diabetes effect size was slightly different from the previous study [43]. First of all, Chen et al.'s study suggested that TG/HDL had no interaction in all age groups (*P* -interaction = 0.3871), which was contrary to our results, which suggested that TG/HDL had interaction in all age groups (*P* -interaction <0.001), and the effect of TG/HDL on diabetes gradually decreased with the increase of age. In addition, they found an inflection point of 1.186, and the HR and 95% CI of the first and second paragraphs were anterior: 1.718 (1.433, 2.060), posterior: 1.409 (0.981, 1.120). This and our results are different. This may differ from the way lost data are handled, they use median interpolation, and we choose multiple interpolation. At the same time, our inflection point is similar to the results of Boizel et al. [44]. They found that the inflection point of TG/HDL in diabetic patients was 1.33. When TG/HDL > 1.33, the size of LDL was more likely to be smaller, which may be related to the degree of LDL oxidation and the mechanism of diabetes.

Research shows that when the TG/HDL ratio increases, free fatty acid in the body will increase, leading to liver triglyceride and low-density lipoprotein. High TG levels are more likely to lead to insulin resistance [45]. When at high TG/HDL level, too much TG binds to insulin receptors, preventing insulin receptors from functioning properly, reduce insulin sensitivity, lead to insulin resistance, and eventually lead to impaired glucose tolerance and diabetes.

Due to the particularity of the data and composition, we cannot guarantee the results of other groups or non-Chinese races nor can we represent specific groups' applicability (pregnant women, children, etc.). At the same time, due to the particularity of the data, diet, exercise, and others can affect the result of residual confusion. Second, we define impaired glucose tolerance as diabetic patients whose fasting blood glucose is greater than or higher than 6.0 mmol/L or self-reported rather than based on the OGTT test or the determination of glycosylated hemoglobin levels, which may be insufficient. In the future, more detailed methods will be needed for more comprehensive follow-up studies.

Although there are some disadvantages, our study has some merits: (1) compared with previous similar studies, our study has a larger sample size and, for the first time, increases the relationship between prediabetes incidence and TG/HDL. (2) Compared with previous studies, our statistical method is more accurate, which solves the nonlinear problem and further analyzes the relationship between TG/HDL and impaired glucose tolerance at different levels. (3) In this study, we analyzed the independent variables from the perspective of both continuous variables and classified variables, which further reduced the contingency in the analysis and increased the results' reliability.

## 5. Conclusion

In conclusion, our study results showed that the increased TG/HDL-C ratio was positively correlated with the risk of prediabetes and diabetes. An elevated TG/HDL-C ratio increases the risk of impaired glucose tolerance in the future, leading to an increased incidence of diabetes. This highlights that lowering the TG/HDL-C ratio may not only reduce the effect on the cardiovascular disease but also contribute to the reduction of the risk of abnormal glucose metabolism. Therefore, monitoring the TG/HDL-C ratio can effectively identify the prediabetic and high-risk diabetic population in the Chinese population, thus helping to warn and control the number of diabetic populations.

## Figures and Tables

**Table 1:** Baseline characteristics of the study participants.

Variables	Total (*n* = 116,855)	Impaired glucose tolerance			Diabetes mellitus		
		Nonimpaired glucose tolerance (*n* = 107,028)	Impaired glucose tolerance (*n* = 9773)	*P* value	Nondiabetes (*n* = 114,682)	Diabetes (*n* = 2173)	*P* value
Age (year), mean ± SD	44.1 ± 12.9	43.2 ± 12.5	54.2 ± 13.0	<0.001	43.9± 12.8	55.7 ± 12.6	<0.001
Gender, *n* (%)							
Male	62,868 (53.8)	56,332 (52.6)	6536 (66.9)	<0.001	61,289 (53.4)	1579 (72.7)	<0.001
Female	53,987 (46.2)	50,750 (47.4)	3237 (33.1)		53,393 (46.6)	594 (27.3)	

Height (cm), mean ± SD	166.3 ± 8.3	166.3 ± 8.3	166.5 ± 8.5	0.124	166.3 ± 8.3	166.9 ± 8.5	<0.001
Weight (kg), mean ± SD	64.9 ± 12.1	64.3 ± 11.9	70.8 ± 12.5	<0.001	64.7 ± 12.0	73.5 ± 13.0	<0.001
BMI (kg/m^2^), mean ± SD	23.3 ± 3.3	23.2 ± 3.2	25.4 ± 3.3	<0.001	23.3 ± 3.3	26.3 ± 3.4	<0.001
FPG (mmol/L), mean ± SD	4.9 ± 0.6	4.9 ± 0.6	5.7 ± 0.7	<0.001	4.9 ± 0.6	6.0 ± 0.7	<0.001
HBP, *n* (%)							
No	99312 (85.0)	92921 (86.8)	6391 (65.4)	<0.001	97999 (85.5)	1325 (61)	<0.001
Yes	17525 (15.0)	14146 (13.2)	3379 (34.6)		16683 (14.5)	848 (39)	

Smoking, *n* (%)							
Current smoker	6672 (20.4)	5878 (19.5)	794 (30.6)	<0.001	19,349 (16.9)	753 (34.7)	<0.001
Ever smoker	1328 (4.1)	1190 (4)	138 (5.3)		3912 (3.4)	123 (5.7)	
Never smoker	24,686 (75.5)	23,022 (76.5)	1664 (64.1)		91,421 (79.7)	1297 (59.7)	

Drinking, *n* (%)							
Current drinker	878 (2.7)	766 (2.5)	112 (4.3)	0.013	2374 (2.1)	89 (4.1)	<0.001
Ever drinker	5535 (16.9)	5069 (16.8)	466 (18)		16,371 (14.3)	411 (18.9)	
Never drinker	26,273 (80.4)	24,255 (80.6)	2018 (77.7)		95,937 (83.7)	1673 (77)	

Cholesterol (mmol/L), median (IQR)	4.7 (4.2, 5.3)	4.7 (4.1, 5.3)	5.0 (4.4, 5.6)	<0.001	4.7 (4.2, 5.3)	5.0 (4.4, 5.7)	<0.001
Triglyceride (mmol/L), median (IQR)	1.1 (0.8, 1.7)	1.1 (0.7, 1.6)	1.6 (1.1, 2.3)	<0.001	1.1 (0.8, 1.6)	1.8 (1.2, 2.6)	<0.001
HDL (mmol/L), median (IQR)	1.4 (1.2, 1.6)	1.4 (1.2, 1.6)	1.3 (1.1, 1.5)	<0.001	1.4 (1.2, 1.6)	1.3 (1.1, 1.5)	<0.001
LDL (mmol/L), median (IQR)	2.7 (2.3, 3.2)	2.7 (2.3, 3.1)	2.8 (2.4, 3.3)	<0.001	2.7 (2.3, 3.2)	2.8 (2.4, 3.3)	<0.001
ALT (U/L), median (IQR)	18.0 (13.0, 27.5)	18.0 (12.9, 27.0)	23.0 (16.2, 35.0)	<0.001	18.0 (13.0, 27.1)	26.0 (18.1, 41.0)	<0.001
AST (U/L), median (IQR)	22.0 (18.7, 26.9)	22.0 (18.4, 26.2)	24.0 (20.0, 29.2)	<0.001	22.0 (18.7, 26.8)	25.0 (21.0, 32.0)	<0.001
CCR (umol/L), median (IQR)	69.6 (58.0, 81.2)	69.1 (57.7, 81.0)	73.0 (62.0, 83.5)	<0.001	69.3 (57.9, 81.0)	73.4 (63.0, 82.9)	<0.001
TG/HDL, median (IQR)	0.8 (0.5, 1.3)	0.8 (0.5, 1.3)	1.2 (0.8, 1.9)	<0.001	0.8 (0.5, 1.3)	1.4 (0.9, 2.2)	<0.001
BUN (mmol/L), median (IQR)	4.6 (3.8, 5.4)	4.5 (3.8, 5.3)	4.9 (4.1, 5.7)	<0.001	4.6 (3.8, 5.4)	4.9 (4.1, 5.8)	<0.001

BMI: body mass index; FPG: fasting blood glucose; HDL: high-density lipoprotein; LDL: low-density lipoprotein; ALT: alanine aminotransferase; AST: aspartate transaminase; CCR: creatinine clearance rate; BUN: blood urea nitrogen.

**Table 2:** Univariate analysis for impaired glucose tolerance and diabetes mellitus.

Variables	Impaired glucose tolerance		Diabetes mellitus	
	HR (95% CI)	*P* value	HR (95% CI)	*P* value
Age	1.05 (1.05, 1.05)	<0.001	1.06 (1.06, 1.06)	<0.001
Gender				
Male	1		1	
Female	0.58 (0.56, 0.61)	<0.001	0.44 (0.4, 0.49)	<0.001

BMI	1.18 (1.17, 1.18)	<0.001	1.24 (1.23, 1.25)	<0.001
FPG	6.05 (5.89, 6.22)	<0.001	10.99 (10.33, 11.68)	<0.001
Cholesterol	1.31 (1.29, 1.34)	<0.001	1.33 (1.28, 1.39)	<0.001
Triglyceride	1.26 (1.25, 1.28)	<0.001	1.27 (1.25, 1.28)	<0.001
HDL	0.6 (0.53, 0.68)	<0.001	0.58 (0.5, 0.66)	<0.001
LDL	1.36 (1.33, 1.4)	<0.001	1.33 (1.25, 1.4)	<0.001
ALT	1.0037 (1.0034, 1.0039)	<0.001	1.0042 (1.0039, 1.0046)	<0.001
AST	1.0054 (1.0048, 1.0061)	<0.001	1.0053 (1.0046, 1.006)	<0.001
BUN	1.21 (1.2, 1.23)	<0.001	1.2 (1.17, 1.24)	<0.001
TG-HDL	1.25 (1.23, 1.26)	<0.001	1.25 (1.24, 1.27)	<0.001
Smoking				
Current smoker	1		1	
Ever smoker	0.84 (0.7, 1.01)	0.33	0.81 (0.67, 0.98)	0.027
Never smoker	0.58 (0.53, 0.63)	<0.001	0.39 (0.36, 0.43)	<0.001

Drinking				
Current drinker	1		1	
Ever drinker	0.55 (0.45, 0.68)	<0.001	0.64 (0.51, 0.8)	<0.001
Never drinker	0.54 (0.44, 0.65)	<0.001	0.48 (0.39, 0.6)	<0.001

Family history of diabetes				
No	1		1	
Yes	1.06 (0.94, 1.2)	<0.001	1.24 (0.98, 1.57)	0.075

BMI: body mass index; FPG: fasting blood glucose; HDL: high-density lipoprotein; LDL: low-density lipoprotein; ALT: alanine aminotransferase; AST: aspartate transaminase; CCR: creatinine clearance rate; BUN: blood urea nitrogen.

**Table 3:** Threshold effect analysis of TG/HDL on incident impaired glucose tolerance.

Outcome	Impaired glucose tolerance	
	HR (95% CI)	*P* value
One-line linear Cox regression model	1.04 (1.02, 1.06)	<0.001
Two-piecewise linear Cox regression model		
TG/HDL < 1.04	1.57 (1.42, 1.73)	<0.001
TG/HDL ≥ 1.04	1.00 (0.98, 1.02)	0.9558
The effect of 2 and 1 is different	0.64 (0.57, 0.71)	<0.0001
Likelihood ratio test	<0.001	

Notes: adjusted for age, gender, BMI, smoking, drinking, family history of diabetes, ALT, AST, BUN, LDL, FPG, and TC.

**Table 4:** Threshold effect analysis of TG/HDL on incident diabetes.

Outcome	Diabetes	
	HR (95% CI)	*P* value
One-line linear Cox regression model	1.16 (1.13, 1.20)	<0.001
Two-piecewise linear Cox regression model		
TG/HDL < 1.33	2.07 (1.80, 2.39)	<0.001
TG/HDL ≥ 1.33	1.08 (1.04, 1.12)	<0.001
The effect of 2 and 1 is different	0.52 (0.45, 0.61)	<0.001
Likelihood ratio test	<0.001	

Notes: adjusted for age, gender, BMI, smoking, drinking, family history of diabetes, ALT, AST, BUN, LDL, FPG, and TC.

**Table 5:** The association between TG/HDL and impaired glucose tolerance in a multiple regression model.

Outcome	Nonadjusted model		Model 1		Model 2		Model 3	
	HR (95% CI)	*P* value	HR (95% CI)	*P* value	HR (95% CI)	*P* value	HR (95% CI)	*P* value
TG/HDL	1.21 (1.20, 1.22)	<0.001	1.10 (1.08, 1.11)	<0.001	1.14 (1.11,1.16)	<0.001	1.04 (1.02, 1.06)	<0.001
<1.04	7.71 (7.05, 8.43)	<0.001	2.37 (2.15, 2.62)	<0.001	2.29 (2.07, 2.53)	<0.001	1.57 (1.42, 1.73)	<0.001
≥1.04	1.19 (1.18, 1.21)	<0.001	1.09 (1.07, 1.10)	<0.001	1.08 (1.07, 1.10)	<0.001	1.00 (0.98, 1.02)	0.9558
TG/HDL group								
<1.04	1		1		1		1	
≥1.04	2.44 (2.34, 2.54)	<0.001	1.43 (1.37, 1.50)	<0.001	1.41 (1.35, 1.47)	<0.001	1.17 (1.12, 1.22)	<0.001

Nonadjusted model: unadjusted; model 1: adjusted for age, gender, and BMI; model 2: model 1 + smoking, drinking, and family history of diabetes; model 3: model 2 + ALT, AST, BUN, LDL, FPG, and TC.

**Table 6:** The association between TG/HDL and diabetes in a multiple regression model.

Outcome	Nonadjusted model		Model 1		Model 2		Model 3	
	HR (95% CI)	*P* value	HR (95% CI)	*P* value	HR (95% CI)	*P* value	HR (95% CI)	*P* value
TG/HDL	1.25 (1.23, 1.26)	<0.001	1.14 (1.12, 1.17)	<0.001	1.14 (1.11, 1.16)	<0.001	1.16 (1.13, 1.20)	<0.001
<1.33	6.60 (5.86, 7.43)	<0.001	2.46 (2.16, 2.81)	<0.001	2.33 (2.04, 2.66)	<0.001	2.07 (1.80, 2.39)	<0.001
≥1.33	1.23 (1.21, 1.25)	<0.001	1.13 (1.10, 1.16)	<0.001	1.12 (1.09, 1.15)	<0.001	1.08 (1.04, 1.12)	<0.001
TG/HDL group								
<1.33	1		1		1		1	
≥1.33	2.96 (2.75, 3.20)	<0.001	1.61 (1.48, 1.74)	<0.001	1.55 (1.43, 1.68)	<0.001	1.52 (1.40, 1.65)	<0.001

Nonadjusted model: unadjusted; model 1: adjusted for age, gender, and BMI; model 2: model 1 + smoking, drinking, and family history of diabetes; model 3: model 2 + ALT, AST, BUN, LDL, FPG, and TC.

## Data Availability

The data used to support the findings of this study can be downloaded from the ‘DATADRYAD' database (http://www.Datadryad.org).

## Abbreviations

BMI: Body mass index
FPG: Fasting blood glucose
HDL: High-density lipoprotein
LDL: Low-density lipoprotein
ALT: Alanine aminotransferase
AST: Aspartate transaminase
CCR: Creatinine clearance rate
BUN: Blood urea nitrogen.
